# Optimization Algorithm for Delay Estimation Based on Singular Value Decomposition and Improved *GCC*-*PHAT* Weighting

**DOI:** 10.3390/s22197254

**Published:** 2022-09-24

**Authors:** Shizhe Wang, Zongji Li, Pingbo Wang, Huadong Chen

**Affiliations:** 1 Academy of Weapony Engineering, Naval University of Engineering, Wuhan 430033, China; 2 Academy of Electronic Engineering, Naval University of Engineering, Wuhan 430033, China

**Keywords:** delay estimation, singular value decomposition, *GCC*-*PHAT*-*ργ* weighting, generalized cross-correlation

## Abstract

The accuracy of time delay estimation seriously affects the accuracy of sound source localization. In order to improve the accuracy of time delay estimation under the condition of low SNR, a delay estimation optimization algorithm based on singular value decomposition and improved *GCC*-*PHAT* weighting (*GCC*-*PHAT*-ργ weighting) is proposed. Firstly, the acoustic signal collected by the acoustic sensor array is subjected to singular value decomposition and noise reduction processing to improve the signal-to-noise ratio of the signal; then, the cross-correlation operation is performed, and the cross-correlation function is processed by the *GCC*-*PHAT*-ργ weighting method to obtain the cross-power spectrum; finally, the inverse transformation is performed to obtain the generalized correlation time domain function, and the peak detection is performed to obtain the delay difference. The experiment was carried out in a large outdoor pool, and the experimental data were processed to compare the time delay estimation performance of three methods: *GCC*-*PHAT* weighting, *SVD*-*GCC*-*PHAT* weighting (meaning: *GCC*-*PHAT* weighting based on singular value decomposition) and *SVD*-*GCC*-*PHAT*-ργ weighting (meaning: *GCC*-*PHAT*-ργ weighting based on singular value decomposition). The results show that the delay estimation optimization algorithm based on *SVD*-*GCC*-*PHAT*-ργ improves the delay estimation accuracy by at least 37.95% compared with the other two methods. The new optimization algorithm has good delay estimation performance.

## 1. Introduction

In recent years, time delay estimation technology [1,2,3,4,5,6,7] has been widely used in navigation and positioning, underwater AUV, radar detection and other fields. Especially in the research of sound source localization, the localization method based on time delay estimation has become the most commonly used and important method due to its low cost and reliable accuracy. The accuracy of time delay estimation directly determines the accuracy of sound source localization.

The generalized cross-correlation method [8,9,10,11,12,13,14] has the advantages of simple principle, high stability, and a small amount of computation when dealing with the delay estimation problem, so it has received extensive attention. However, this method has poor anti-noise ability. As the signal-to-noise ratio decreases, the peak value of the correlation function is no longer sharp, and the performance of the delay estimation drops sharply. However, there will be large noise interference in many practical scenarios, so the noise reduction processing is particularly important.

Based on the above research, it can be seen that there are two keys to improving the accuracy of delay estimation: one is how to effectively reduce noise to improve the signal-to-noise ratio; the other is how to improve the weighting function to make the correlation peak sharper. Reference [15] introduces an improved wavelet threshold function for signal denoising and reconstruction, and uses the GCC method for delay estimation. This method overcomes the shortcomings of traditional soft and hard threshold functions and improves the performance of delay estimation. However, based on the wavelet threshold, the denoising of the signal is restricted by factors such as wavelet base selection and decomposition scale. Reference [16] proposed a data processing method combining wavelet packet noise reduction algorithm and EMD decorrelation algorithm, which can effectively suppress modal aliasing and white noise interference at the same time, but lacks the means of suppressing impulse noise. Reference [17] used the cepstral method to separate the glottal impulse mechanism and the vocal tract response, and combined the spectral subtraction method to design an effective scheme to distinguish the useful frequency bands of noisy signals. Finally, using the spectral characteristics of the eigenmode function after empirical mode decomposition and signal reconstruction, this method significantly improves the anti-noise performance of the delay estimation algorithm, but the algorithm is complex, the application scenario is single, and performance testing in different scenarios is required.

The singular value decomposition method has the advantages of an obvious denoising effect and strong stability. Using this method for signal preprocessing can not only preserve the signal deformation information, but also better filter out the noise pollution. The collected sound source signals are mixed with various environmental noise interference signals. Therefore, the singular value decomposition is to construct a matrix that also contains noise, and the elements in the non-zero singular value matrix represent the distribution of signal and noise energy concentration. Large non-zero singular values represent useful signals, and small ones are interfering noise. The purpose of eliminating noise can be achieved by zeroing the smaller one. For underwater shock signals, the energy of the useful signal segment is relatively high. When the singular value decomposition method is used for noise reduction, the energy distribution of the signal represented by the elements in the non-zero singular value matrix and the noise are obviously different. Therefore, the singular value decomposition method can obtain a good noise reduction effect.

In sound source localization, the accuracy of time delay estimation is very important, which plays a decisive role in the final localization accuracy. Therefore, improving the accuracy of time delay estimation is of great significance to improve the accuracy of sound source localization. However, in some actual measurement environments, the collected acoustic signal contains a lot of noise information, which will have a great impact on the accuracy of time delay estimation. Therefore, it is very important to preprocess the signal with noise reduction first. Secondly, in the time-delay estimation algorithm, because there may be some correlation between noise and noise in the measured signal and between the target signal and noise, the traditional cross-correlation time-delay estimation method based on correlation analysis has difficulty obtaining a more accurate time-delay estimation value, and it is also of great significance to optimize the time-delay estimation method.

The papers [18,19] study the generalized cross-correlation time-delay estimation method based on singular value decomposition, and [20] studies the generalized cross-correlation time-delay estimation method based on improved Phat weighting, which can improve the time-delay estimation accuracy.

This paper combines the two and proposes a time delay estimation optimization algorithm based on singular value decomposition noise reduction. The method first uses singular value decomposition to preprocess the signal to improve the signal-to-noise ratio; secondly, it uses the *GCC-PHAT-*ργ weighting function to process the cross power spectrum and sharpen the correlation peak to improve the accuracy of delay estimation.

## 2. Theoretical Introduction of Delay Estimation Optimization Algorithm

### 2.1. TDOA Signal Model

In the actual scenario of positioning based on TDOA delay estimation, the signal model [21,22,23] can be expressed as:

(1) $$x_{1}\left(n\right)=s\left(n\right)+n_{1}\left(n\right)$$

(2) $$x_{2}\left(n\right)=As\left(n-D\right)+n_{2}\left(n\right)$$

where s(n) is the target signal, $x_{1}\left(n\right)$ and $x_{2}\left(n\right)$ are the two acquisition signals, and $n_{1}\left(n\right)$ and $n_{2}\left(n\right)$ are noise. Assuming that the noise and the target signal are uncorrelated, *D* is the time delay and *A* is the attenuation factor.

### 2.2. Singular Value Decomposition Noise Reduction Theory

The singular value decomposition [24,25,26,27,28,29,30,31,32] method is referred to as the SVD method, which is an important matrix decomposition method when dealing with mathematical problems. The noise reduction principle is to decompose the matrix on the basis of phase space reconstruction, set the singular value corresponding to the noise to zero, and then use the inverse operation to reconstruct the signal, so as to achieve the purpose of noise reduction.

Select the Hankel matrix as the trajectory matrix of singular value decomposition, set the noisy signal X=[x(1),x(2),⋯x(N)], construct the Hankel matrix $A_{m\times n}$:

(3) $$A=\left[\begin{array}{cccc} x(1) & x(2) & \cdots & x(n)\\ x(2) & x(3) & \cdots & x(n+1)\\ \vdots & \vdots & \; & \vdots \\ x(m) & x(m+1) & \cdots & x(N) \end{array}\right]$$

The value range of n is N/20−N/2, m+n−1=N, and *n* is taken as N/2 in this paper. Matrix *A* is an m×n matrix with rank *r*. There are m×m orthogonal matrix *U* and n×n orthogonal matrix *V*, so that $A=U\Lambda V^{T}$.

(4) $$\Lambda =\left[\begin{array}{cc} \Sigma & 0\\ 0 & 0 \end{array}\right]=\left[\begin{array}{ccccccc} \delta_{1} & 0 & 0 & \cdots & \cdots & \cdots & 0\\ 0 & \delta_{2} & \; & & \; & & 0\\ 0 & \; & \ddots & \; & & \; & 0\\ \vdots & \; & & \delta_{r} & \; & & \vdots \\ \vdots & \; & & \; & 0 & \; & \vdots \\ \vdots & \; & & \; & & \ddots & \vdots \\ 0 & 0 & 0 & \cdots & \cdots & \cdots & 0 \end{array}\right]$$

The diagonal elements of matrix Λ are singular values of matrix *A*, and it satisfies $\delta_{1}\gg \delta_{2}\gg \cdots \gg \delta_{r}>0$, where different singular values reflect different energy concentrations. The law of singular values corresponding to different signals is analyzed, the singular value corresponding to noise is set to zero, the appropriate singular value is selected to reconstruct the signal, and the effective signal can be restored to achieve the purpose of denoising. There are three ways to select singular values:

(1) Singular value difference spectrum method

After the Hankel matrix is decomposed, $\delta_{1}$, $\delta_{2}$, ⋯, $\delta_{r}$ are formed. If the first *k* singular values are significantly larger than the last r−k singular values, that is, the singular value mutates at the *k*-th point, the first *k* singular values are the ideal signals to be extracted.

(5) $$b_{i}=\delta_{i}-\delta_{i+1},i=1,2,\cdots ,r-1$$

The $b_{i}$ sequence is called the difference spectrum of singular values. The difference spectrum can effectively and automatically determine the maximum mutation point $b_{k}$, set the following $r_{k}$ singular values to zero, reconstruct the matrix, and realize the noise reduction of the signal.

(2) Feature mean method

The singular values $\delta_{1}$, $\delta_{2}$, ⋯, $\delta_{r}$ obtained by the decomposition of the Hankel matrix are the square roots of the eigenvalues $\lambda_{1}$, $\lambda_{2}$, ⋯, $\lambda_{r}$ of the square matrix $AA^{T}$. The eigenvalues smaller than the mean of all eigenvalues are set to zero, and the matrix is reconstructed to realize the denoising of the signal.

(6) $$\delta_{i}=\sqrt{\lambda_{i}},i=1,2,\cdots ,r$$

(3) Singular value median method

Arrange the singular values $\delta_{1}$, $\delta_{2}$, ⋯, $\delta_{r}$ obtained by the decomposition of the Hankel matrix in ascending or descending order, obtain the median value of the sequence, set the eigenvalues less than the median value of all singular values to zero, reconstruct the matrix, and realize the noise reduction of the signal.

In the following, for the same noise-containing signal, the singular value difference spectrum method, the characteristic mean method and the median method are used for processing, to compare the noise reduction effect. The simulated signal source is: x=sin3πt·cos10πt+sin(20πt+sin30πt)+α(n), where α(n) is Gaussian white noise with variance 0.3 and mean 0, and the sampling time is 0.005 s. The mean square error and the signal-to-noise ratio are used to measure the noise reduction effect, and the definitions are as shown in Equations (7) and (8). Among them, *x* is the actual signal, *y* is the signal after noise reduction, and *n* is the signal length. The difference in the noise reduction effect of the three methods is shown in Table 1.

(7) $$\mathrm{RMSE}=\sqrt{\frac{\sum {\left(x_{i}-y_{i}\right)}^{2}}{n}}$$

(8) $$\mathrm{SNR}=10\times lg\frac{\sum x_{i}^{2}}{\sum {\left(x_{i}-y_{i}\right)}^{2}}$$

**Figure 1:**
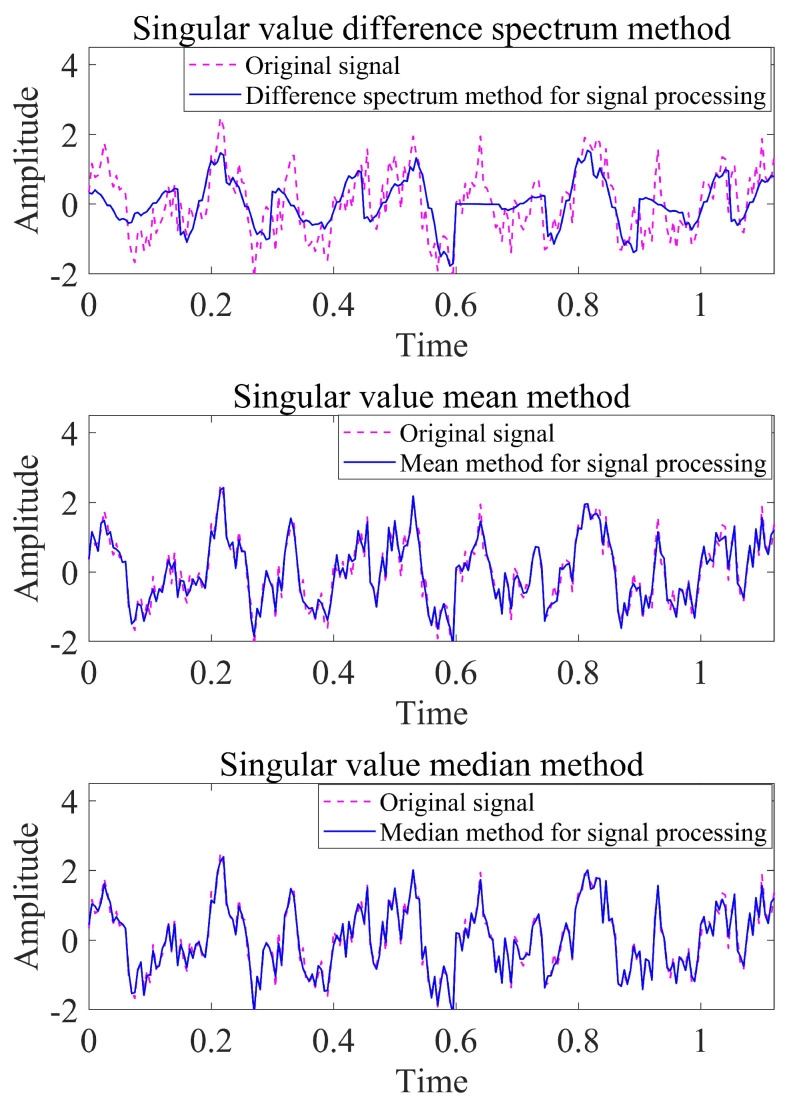
Effect diagram of three methods of noise reduction.

It can be seen from Table 1 that the singular value median method has the highest SNR of 16.3440 and the smallest mean square error of 0.1429. The singular value median method has a significant denoising effect, and can restore the original characteristics of the signal well, and the noise reduction effect is better than the other two methods. Figure 1 shows the effect of noise reduction processing using three methods, respectively.

### 2.3. Improved PHAT Weighted Generalized Cross-Correlation Delay Estimation Algorithm

The *GCC-PHAT* weighting method [12,33,34,35,36] can achieve better delay estimation results when the signal-to-noise ratio is high and the reverberation is weak. This leads to false peaks and increases the delay estimation error. In order to solve this problem, the parameter ρ is introduced, and the value of ρ is determined according to the signal-to-noise ratio in the actual environment. On the other hand, because the signal energy is small under the condition of low signal-to-noise ratio, using *GCC*-*PHAT* weighting will make the denominator of the weighting function approach 0, which will bring greater error, so consider adding a coherence factor $\gamma_{12}\left(\omega \right)$ to the denominator of the weighting function. The new weighting function is given below:

(9) $$\psi_{12}\left(\omega \right)=\frac{1}{{\left|G_{12}\left(\omega \right)\right|}^{\rho }+\left|\gamma_{12}^{2}\left(\omega \right)\right|},0\leq \rho \leq 1$$

In the formula, $\gamma_{12}\left(\omega \right)=\frac{G_{12}\left(\omega \right)}{\sqrt{G_{11}\left(\omega \right)G_{22}\left(\omega \right)}}$ is the coherence factor, $G_{11}\left(\omega \right)$ is the self-power spectrum of the signal $x_{1}\left(t\right)$, $G_{22}\left(\omega \right)$ is the self-power spectrum of the signal $x_{2}\left(t\right)$, $G_{12}\left(\omega \right)$ is the cross-power spectrum of the signals $x_{1}\left(t\right)$ and $x_{2}\left(t\right)$.

## 3. Principle of Delay Estimation Optimization Algorithm

**Figure 2:**
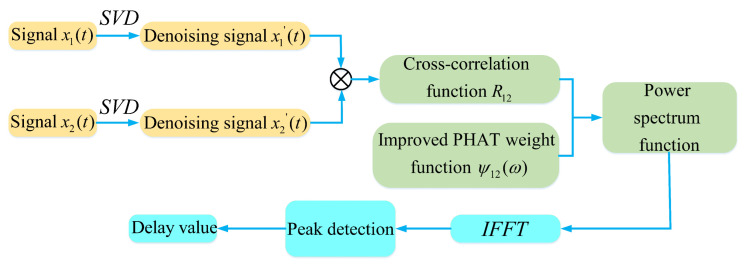
Flow chart of improved time delay estimation algorithm.

The flowchart of the improved time delay estimation algorithm is shown in Figure 2. The calculation process of the improved time delay estimation algorithm is as follows:

(1) Performing singular value decomposition noise reduction processing on the original signals $x_{1}$ and $x_{2}$ to obtain denoised signals $x_{1}^{\prime }$ and $x_{2}^{\prime }$;
(2) Performing cross-correlation on the denoised signals $x_{1}^{\prime }$ and $x_{2}^{\prime }$ to obtain the cross-correlation function $R_{12}$;
(3) Using the improved PHAT weighting function to process the cross-correlation function, the power spectrum function is obtained;
(4) Performing inverse Fourier transform on the power spectrum function to obtain the generalized cross-correlation time-domain function;
(5) Performing peak detection on the generalized cross-correlation time-domain function to obtain the delay difference.

## 4. Simulation Analysis of Analog Signals

**Figure 3:**
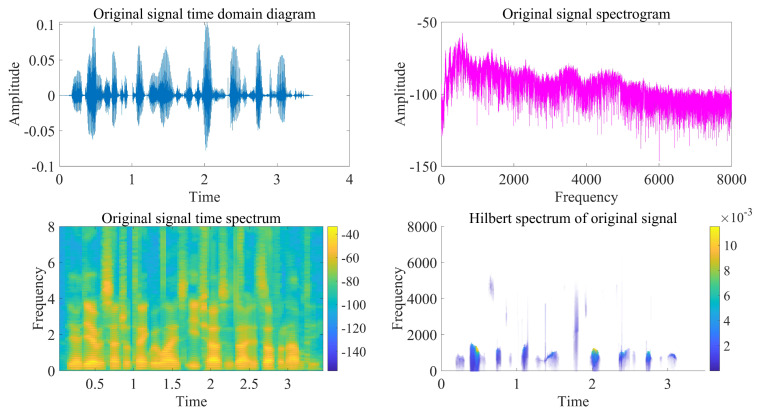
Original signal time-frequency analysis.

**Figure 4:**
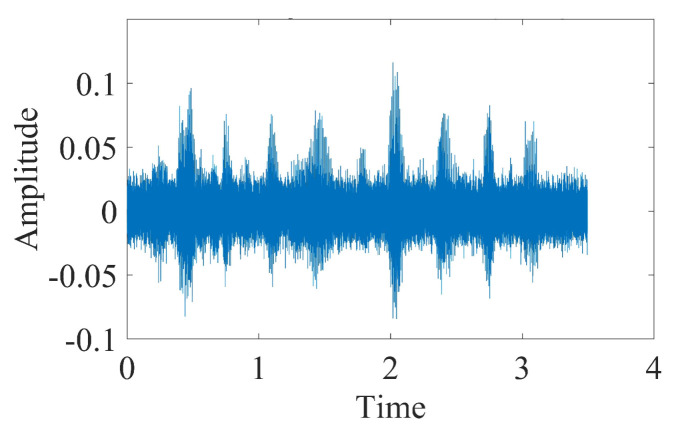
Time domain figure of noisy original signal.

**Figure 5:**
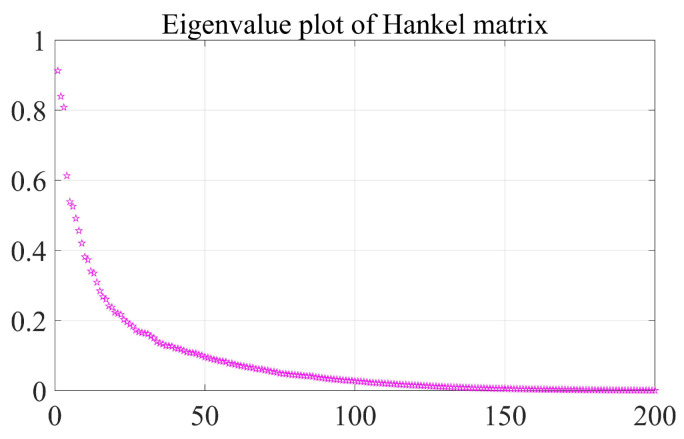
Eigenvalue figure of Hankel matrix.

**Figure 6:**
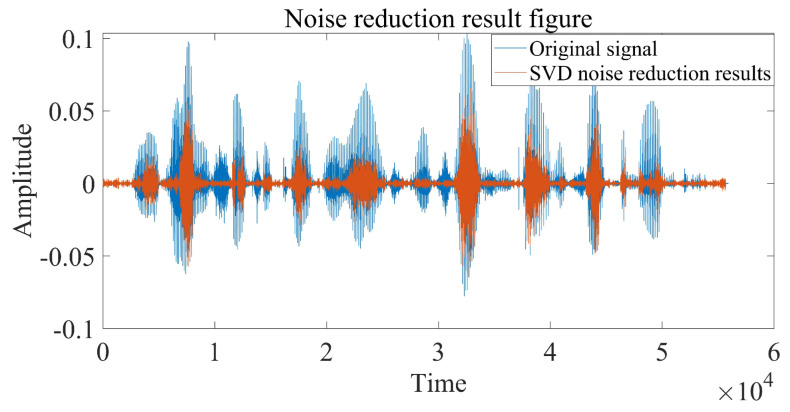
Comparison of SVD noise reduction results and original signals.

This chapter uses the test library speech signal numbered MSJS1-SI869.WAV in the TIMIT standard library as the sound source signal, the signal sampling frequency is 16,000 Hz, and Gaussian white noise is added manually to verify the delay estimation accuracy of the optimization algorithm in this paper. The time-domain diagram, spectrogram, time-frequency spectrum and Hilbert spectrum of the MSJS1-SI869 test library speech signal are shown in Figure 3. After manually adding Gaussian white noise, SNR = 0, and the time-domain diagram of the noisy signal is shown in Figure 4. The singular value median method is used to process the noisy signal, the eigenvalues of the Hankel matrix are shown in Figure 5, and the comparison between the noise reduction result and the original signal is shown in Figure 6.

**Figure 7:**
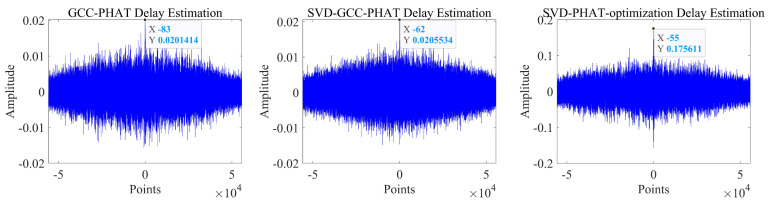
Comparison of delay estimation peak values of three methods when SNR = −20.

**Figure 8:**
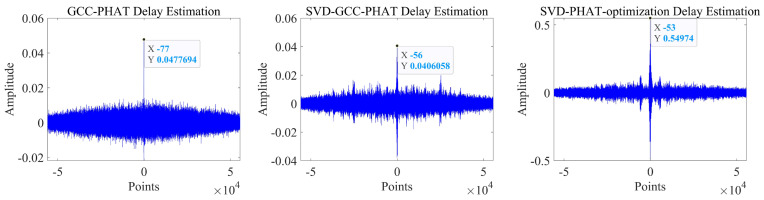
Comparison of delay estimation peak values of three methods when SNR = −10.

**Figure 9:**
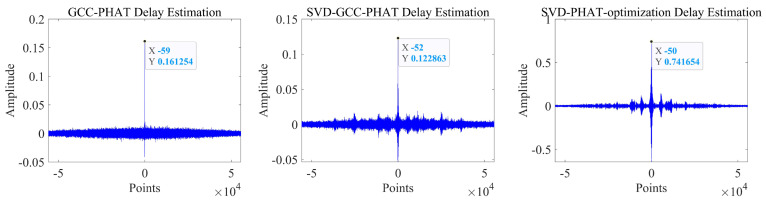
Comparison of delay estimation peak values of three methods when SNR = 0.

**Figure 10:**
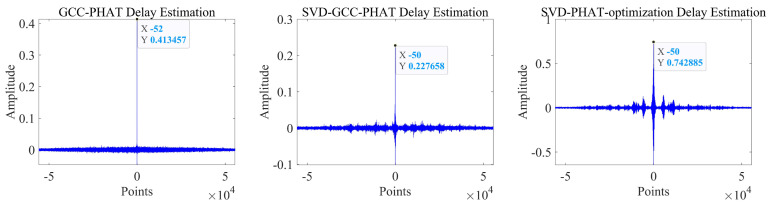
Comparison of delay estimation peak values of three methods when SNR = 10.

**Figure 11:**
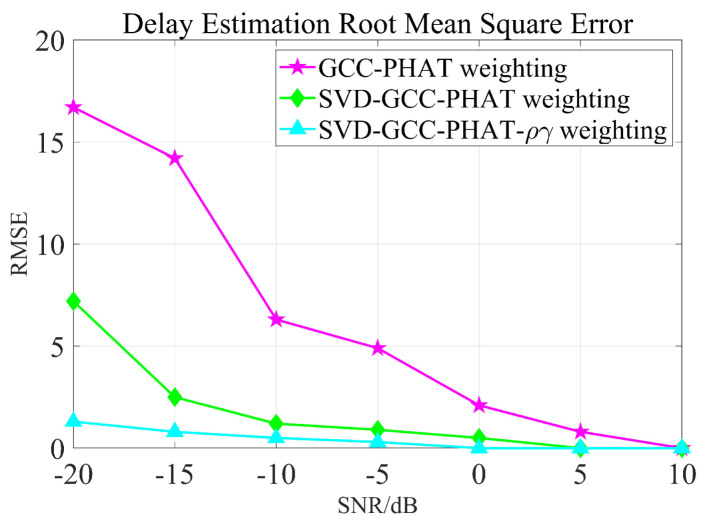
RMSE of three methods for delay estimation.

It can be seen that the noise reduction effect of the singular value median method is obvious, and the signal-to-noise ratio is greatly improved. In order to verify the time delay estimation performance of the three methods of *GCC-PHAT* weighting, *SVD-GCC-PHAT* weighting and *SVD-GCC-PHAT*-ργ weighting, the original signal was manually delayed by 50 sampling points and then Gaussian white noise was added. The signal-to-noise ratio variation range is −20 dB–10 dB. The results are shown in Figure 7, Figure 8, Figure 9 and Figure 10. The abscissa represents the number of delay points, and the ordinate represents the degree of correlation. Fifty random experiments were performed, and Figure 11 shows the root mean square error curves of the three methods under different signal-to-noise ratio conditions.

It can be seen from Figure 7, Figure 8, Figure 9 and Figure 10 that under the condition of high signal-to-noise ratio, *GCC-PHAT* weighting, *SVD-GCC-PHAT* weighting and *SVD-GCC-PHAT-*ργ weighting can all accurately estimate the delay difference. However, as the signal-to-noise ratio decreases, interference gradually appears around the weighted peak of *GCC-PHAT*, the correlation peak is no longer sharp, and even false peaks appear, resulting in a large delay estimation error. The weighted estimation accuracy of *SVD-GCC-PHAT* also gradually deviates, but compared with the *GCC-PHAT* weighting method, the singular value decomposition method is used to denoise the original signal, and the delay estimation error is lower than that of the *GCC-PHAT* weighting method. The *SVD-GCC-PHAT*-ργ weighting not only uses *SVD* to denoise the signal, but also improves the weighting function, which strengthens the useful components in the signal and sharpens the correlation peak. A more accurate delay difference value is obtained, which shows certain advantages. It can also be seen from Figure 11 that as the SNR increases, the delay estimation accuracy of the three methods improves, but under the condition of low SNR, the delay estimation of the *SVD-GCC-PHAT*-ργ weighting method has more superior performance.

## 5. Experiment and Performance Analysis

### 5.1. Experimental System Construction

In this chapter, the actual pool experiment was carried out, and the measured signal was analyzed and processed to verify the reliability of the method in this paper.

**Figure 12:**
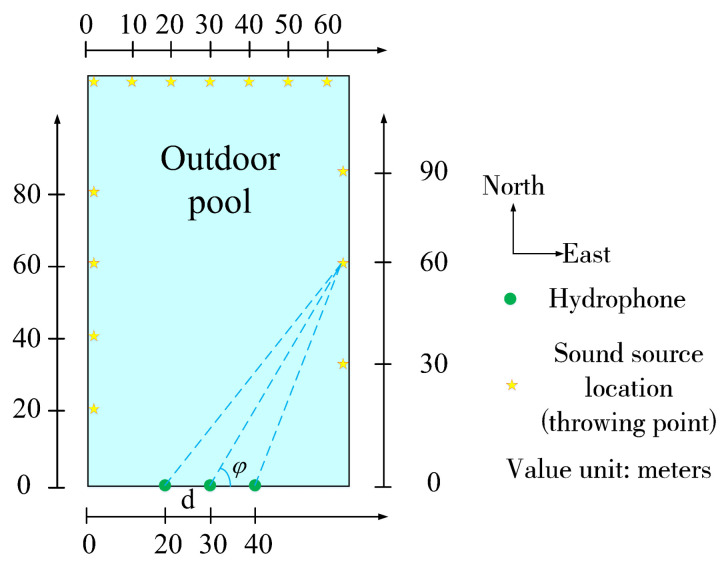
Schematic diagram of experimental layout.

**Figure 13:**
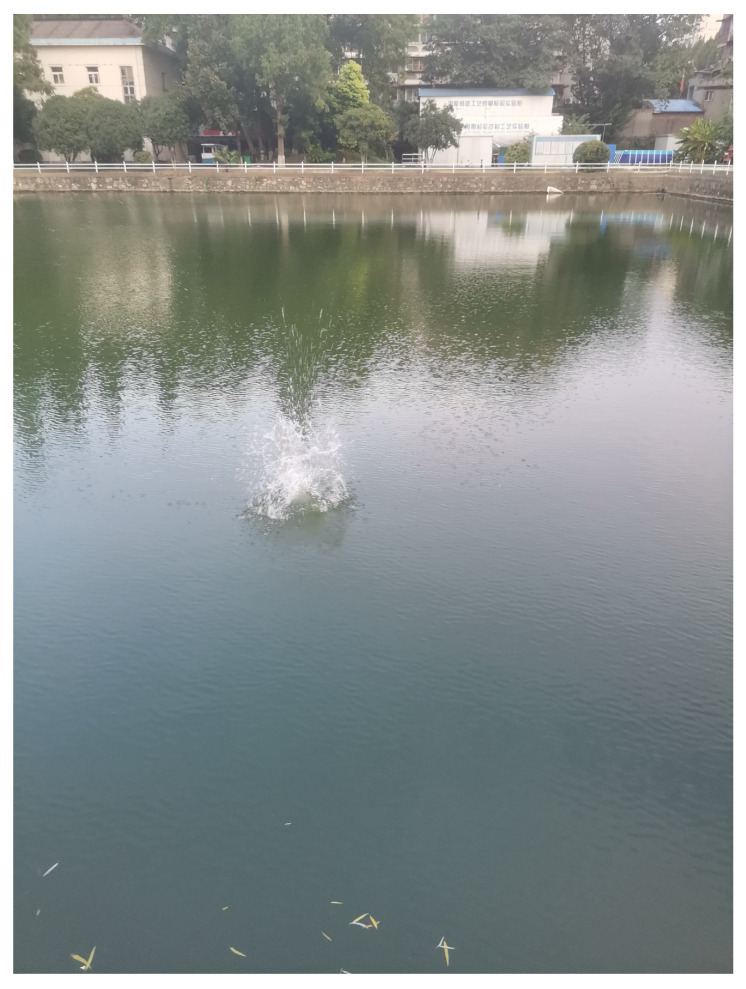
Experimental site diagram.

The experiment was carried out in a larger outdoor pool, which was about 120 m long, 70 m wide and 3 m high. A three-element linear acoustic sensor array with an array element spacing of 5 m is arranged on the wall of a broad side of the pool, and the depth of the three hydrophones is about 2 m. The underwater sound signal is generated by throwing heavy objects at different points in the pool, the schematic diagram of the experimental arrangement is shown in Figure 12, the experimental site is shown in Figure 13. The sensitivity of the acoustic sensor is less than −200 dB when detecting the sound source in the frequency range of 10–50,000 Hz, the sampling frequency of the acquisition instrument is 128 kHz, and each acquisition time is set to 10 s. The day of the experiment was sunny, breezy, and the water surface of the pool was calm.

### 5.2. Experimental Signal Processing

The collected acoustic signals are analyzed according to the following steps:

(1) Firstly, time-frequency domain analysis is performed to analyze the characteristics of the acquired signal;
(2) Then, the singular value decomposition process is performed to obtain the denoised signal, and the difference between the acquired signal and the denoised signal is compared, and the reliability and correctness of the singular value decomposition noise reduction are verified by the frequency domain analysis of the denoised signal;
(3) Finally, based on the *GCC-PHAT-*ργ-weighted delay estimation algorithm, the three-way signal is estimated for pairwise delays, and the delay difference is obtained, which is compared with the theoretical delay difference.

Taking the acoustic signal emitted at a position near (120 m, 90°) as an example, analyze and process the signal according to the above steps.

**Figure 14:**
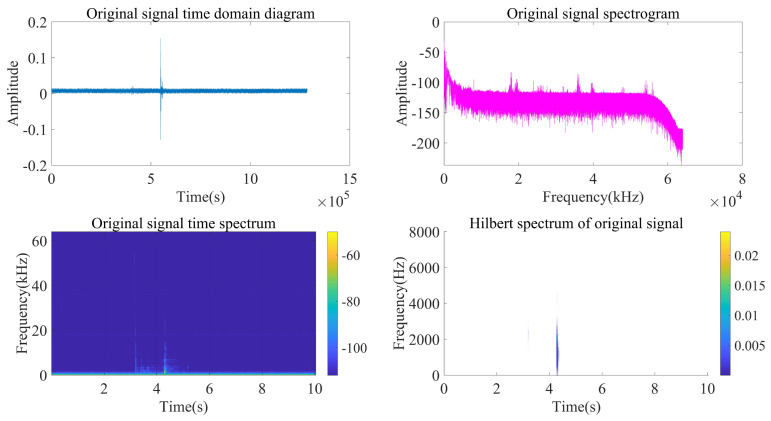
First-channel acquisition signal processing and analysis.

**Figure 15:**
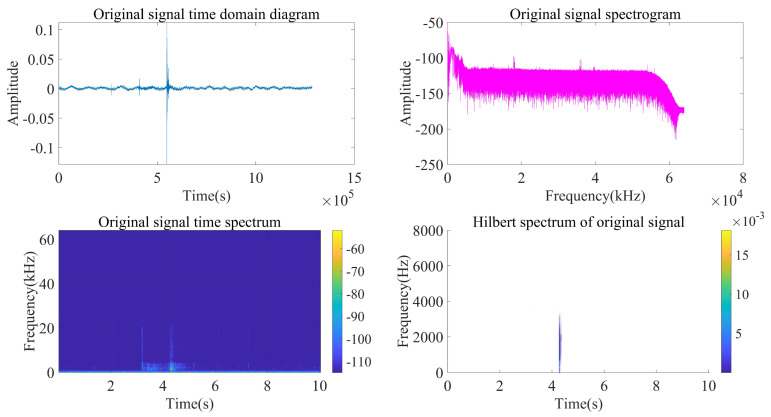
Second-channel acquisition signal processing and analysis.

**Figure 16:**
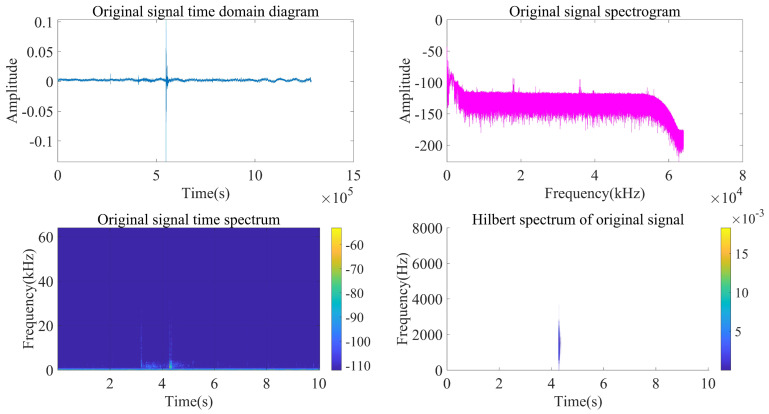
Third-channel acquisition signal processing and analysis.

**Figure 17:**
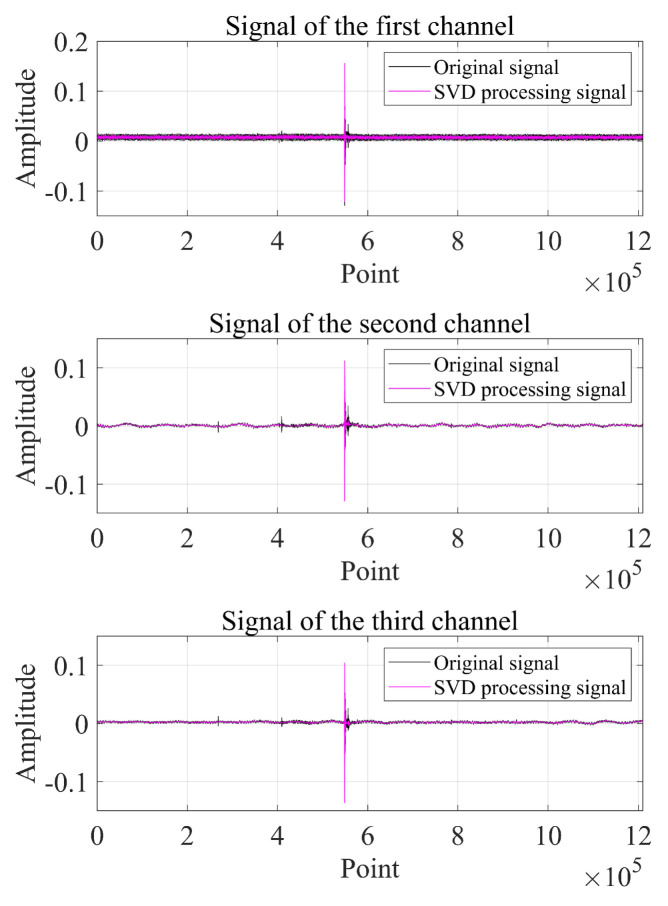
Comparison of the original value of the three-channel signal and the noise reduction value.

**Figure 18:**
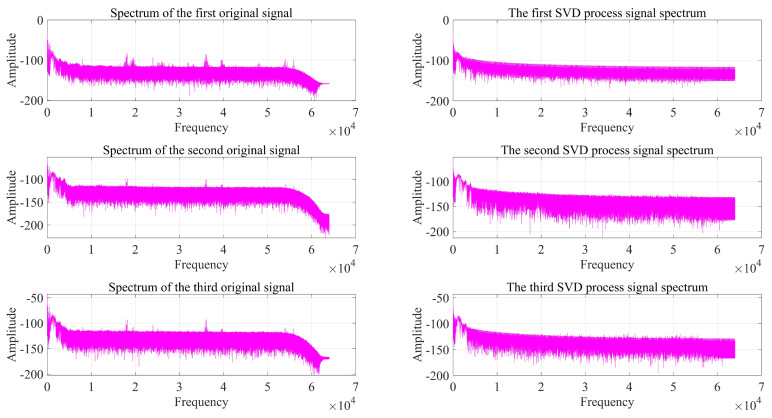
Comparison of the original spectrum of the three-channel signal and the spectrum after noise reduction.

As can be seen from Figure 14, Figure 15 and Figure 16, the impact signal generated by throwing heavy objects into the pool in the three-way signal is very obvious, occurring between 4–5 s. The singular value median method is used for noise reduction processing. Figure 17 is a comparison effect diagram of the original value of the three-way signal and the noise reduction processing value. Figure 18 is a comparison effect diagram of the original spectrum of the three-channel signal and the spectrum after noise reduction.

It can be seen from Figure 17 that the denoising effect of the singular value median method is obvious, which not only removes the interference of noise, but also fully retains the characteristics of the shock signal, which is beneficial to the subsequent delay estimation. It can be seen from Figure 18 that the signal after noise reduction by the singular value median method almost completely retains the characteristics of the effective signal, and there is no distortion phenomenon. Figure 17 and Figure 18 both verify the reliability and correctness of singular value decomposition noise reduction processing of the shock signal.

### 5.3. Algorithm Verification and Analysis

In order to further verify the important role of the research in this paper in improving the accuracy of delay estimation, the acoustic signal in the previous section is analyzed, and the delay estimation performance of the three methods is compared: direct generalized cross-correlation (*GCC-PHAT*) weighting, generalized cross-correlation (*SVD-GCC-PHAT*) weighting after SVD processing, and improved generalized cross-correlation (*SVD-GCC-PHAT-*ργ) weighting after SVD processing. For the improved generalized cross-correlation weighting method after SVD processing (*SVD-GCC-PHAT-*ργ), the value range of ρ is 0 to 1, and its value is related to the signal-to-noise ratio of the measured environment. In this paper, the selection of a value of ρ is as follows: set a certain step, traverse ρ from 0 to 1, and select the value of ρ corresponding to the smallest delay estimation error as the final value.

The delay estimation results are shown in Table 2. The performance evaluation index is the number of delay points.

**Figure 19:**
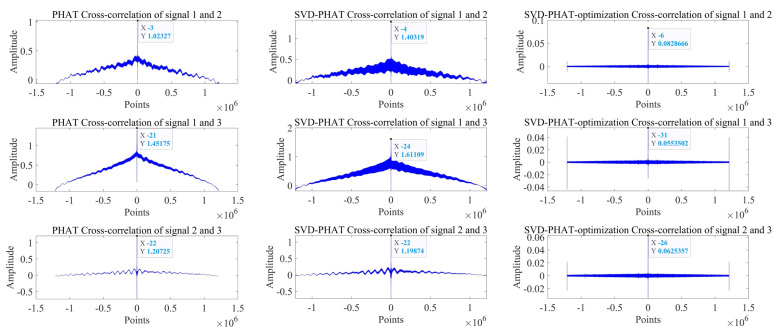
Delay estimation performance of *GCC-PHAT* weighting, *SVD-GCC-PHAT* weighting and *SVD-GCC-PHAT-*ργ weighting.

It can be seen from Figure 19 that as the signal undergoes singular value noise reduction processing and weighting function improvement, the peak sharpness of the correlation function increases significantly, the resolution becomes more and more accurate, and the accuracy of delay estimation is greatly improved.

Compared with the direct PHAT weighted delay estimation method (*GCC-PHAT*), the delay estimation method based on singular value decomposition noise reduction and improved PHAT weighting function (*SVD-GCC-PHAT-*ργ) improves the estimation accuracy of $\tau_{12}$, $\tau_{13}$ and $\tau_{23}$. They were increased by 47.85%, 62.38%, and 69.44%, respectively. Compared with the time delay estimation method based on singular value decomposition noise reduction and PHAT weighting (*SVD-GCC-PHAT*), $\tau_{12}$, $\tau_{13}$ and $\tau_{23}$ estimation accuracy increased by 37.95%, 53.72%, and 69.44%, respectively. The improvement of time delay estimation accuracy fully demonstrates the significance of singular value noise reduction and improved PHAT weighting function.

## 6. Conclusions

In this paper, an optimization algorithm for delay estimation based on singular value decomposition and *GCC-PHAT-*ργ weighting is proposed. The proposed delay estimation method improves anti-noise performance and sharpens correlation peaks. The time delay estimation performance and anti-noise performance of the three methods are verified through the analysis and processing of the simulated signal. By analyzing and processing the measured outdoor data, and comparing the three methods based on *GCC-PHAT* weighting, *SVD-GCC-PHAT* weighting and *SVD-GCC-PHAT*-ργ weighting, the accuracy of delay estimation of the new algorithm in this paper is verified. Compared with the time-delay estimation method based on basic correlation analysis, this method does not greatly increase the calculation amount, but the accuracy of time-delay estimation is improved, the research in this paper can be applied to the actual scene of TDOA sound source localization, which is of great significance for target detection, localization and tracking based on time delay estimation, and has certain application prospects.

## Figures and Tables

**Table 1:** Comparison of noise reduction effects of three methods.

Method	RMSE	SNR
Singular value difference spectrum method	0.6722	2.8962
Feature mean method	0.2487	11.5337
Singular value median method	0.1429	16.3440

**Table 2:** Comparison of results of different time delay estimation methods.

Method	$\tau_{12}\;\left(\mathrm{Points}\right)$	$\tau_{13}\;\left(\mathrm{Points}\right)$	$\tau_{23}\;\left(\mathrm{Points}\right)$
Theoretical delay value	−9.27	−37.03	−27.76
*GCC-PHAT* weighting	−3	−21	−22
*SVD-GCC-PHAT* weighting	−4	−24	−22
*SVD-GCC-PHAT-*ργ weighting	−6	−31	−26

## Data Availability

Not applicable.
